# Serological Evidence of Japanese Encephalitis Virus Circulation in Asian Children From Dengue-Endemic Countries

**DOI:** 10.1093/infdis/jiy513

**Published:** 2018-08-28

**Authors:** Joshua Nealon, Anne-Frieda Taurel, Sutee Yoksan, Annick Moureau, Matt Bonaparte, Luong Chan Quang, Maria R Capeding, Ari Prayitno, Sri Rezeki Hadinegoro, Danaya Chansinghakul, Alain Bouckenooghe

**Affiliations:** 1 Sanofi Pasteur, Singapore; 2 Mahidol University, Nakhonpathom, Thailand; 3 Sanofi Pasteur, Marcy L’Etoile, France; 4 Sanofi Pasteur, Swiftwater, Pennsylvania; 5 Pasteur Institute Ho Chi Minh City, Vietnam; 6 Research Institute for Tropical Medicine, Alabang, Muntinlupa City, Philippines; 7 Universitas of Indonesia, Jakarta; 8 Sanofi Pasteur, Bangkok, Thailand

**Keywords:** epidemiology, flavivirus, encephalitis, Japanese, seroepidemiologic studies

## Abstract

**Background:**

Japanese encephalitis virus (JEV) is a zoonotic, mosquito-borne flavivirus, distributed across Asia. Infections are mostly mild or asymptomatic, but symptoms include neurological disorders, sequelae, and fatalities. Data to inform control strategies are limited due to incomplete case reporting.

**Methods:**

We used JEV serological data from a multicountry Asian dengue vaccine study in children aged 2–14 years to describe JEV endemicity, measuring antibodies by plaque reduction neutralization test (PRNT_50_).

**Results:**

A total 1479 unvaccinated subjects were included. A minimal estimate of pediatric JEV seroprevalence in dengue-naive individuals was 8.1% in Indonesia, 5.8% in Malaysia, 10.8% in the Philippines, and 30.7% in Vietnam, translating to annual infection risks varying from 0.8% (in Malaysia) to 5.2% (in Vietnam). JEV seroprevalence and annual infection estimates were much higher in children with history of dengue infection, indicating cross-neutralization within the JEV PRNT_50_ assay.

**Conclusions:**

These data confirm JEV transmission across predominantly urban areas and support a greater emphasis on JEV case finding, diagnosis, and prevention.

## Teaser

Japanese encephalitis virus circulation has been demonstrated in urban areas of Indonesia, Malaysia, the Philippines, and Vietnam. Serological data indicate that up to 5% of children are infected annually.

Japanese encephalitis virus (JEV) is a mosquito-borne flavivirus, distributed across endemic or epidemic-prone countries in East, Southeast, and South Asia. Extending from North Korea, southeastern Russia, Japan, and Northern China to Western Pacific islands including the Philippines, Papua New Guinea, and the far north of Australia, and west to India and southern Pakistan: >3 billion people are at risk of infection [1]. A variety of vertebrate hosts sustain transmission in zoonotic cycles with mosquitoes, predominantly *Culex tritaeniorhynchus* [2]. Humans are infected as incidental, dead-end hosts and may be at particular risk when in proximity to pigs and ardeid birds, which experience durations and levels of viremia capable of infecting vector mosquitoes [2, 3].

After humans are bitten by an infected mosquito, the virus is thought to amplify in the cells of the peripheral lymphatic system causing a transient and mostly low-grade viremia for ~1 week. Although infections are common in endemic areas, most are either asymptomatic or resolve after acute undifferentiated fever and are unlikely to be diagnosed as Japanese encephalitis [2, 4]. Estimates of the proportion of infections that lead to symptomatic disease vary widely from 1:25 to 1:1000 [5]. Estimates of the proportion of symptomatic disease are higher in studies from non-indigenous US servicemen in Asia than indigenous populations, perhaps a consequence of (1) viral or human genetics, (2) level of health, (3) immune status, (4) more sensitive surveillance, and (5) increased laboratory confirmation [6–8].

A small proportion of infections proceed to more severe disease after invasion of the central nervous system, leading to an encephalitis; this results in a broad range of neurological disorders including convulsions, prolonged seizures, respiratory abnormalities, and spasms [9]. In hospitalized individuals, approximately 30% will die, and approximately 50% of survivors will suffer severe residual neurological disease [9, 10].

Although considered rare, Japanese encephalitis cases cause significant morbidity and mortality with an estimated 67900 incident symptomatic cases per year across affected countries [11]. Even severe cases may be unreported to public health authorities due to a combination of low level of clinical suspicion, infrequent use of laboratory confirmation, and a wide spectrum of symptoms [9, 12]. Several licensed vaccines are available, and vaccination is recommended both for those living in and traveling to endemic areas. Underrecognition of disease contributes to undervaccination [1, 13].

In the absence of reliable incidence data, seroepidemiological methods can be used to measure exposure and make inferences around endemicity of diseases [2, 14]. Age reflects duration of exposure, and because JEV antibodies persist for life, age-stratified data describe the proportion of individuals historically infected, from which the infection rate can be calculated [15]. A challenge in this approach stems from the specificity of diagnostics that have well documented cross-reactivity between members of the flavivirus family [16]. Assays detecting immunoglobulin G antibodies, raised after recent vaccination or recent wild-type infection, are particularly cross-reactive, and a positive result cannot be considered specific in areas where multiple flaviviruses cocirculate [17, 18]. Cross-reactivity typically decreases as the immune response evolves from an initial more heterotypic to a homotypic response. Neutralizing assays including the plaque reduction neutralization test (PRNT), in which the dilution of serum required to neutralize live viruses is quantified, are more specific and are considered the gold standard in detecting historical flavivirus exposure [19]. For JEV, a PRNT titer ≥1:10 dilution by PRNT_50_ is considered protective from infection; a more stringent threshold, PRNT_90_, may be preferred for epidemiological studies of historical exposure, reducing the risk of background serum cross-reactivity [20, 21].

CYD14 (ClinicalTrials.gov number NCT01373281) was an observer-blinded dengue vaccine study conducted in 2011–2017 in 10275 children aged 2–14 years in Indonesia, Malaysia, Thailand, the Philippines, and Vietnam [22]. From an immunological study subset, JEV seroprevalence was ascertained by PRNT at the study start before any vaccines were administered. Sites were urban, selected based on their high dengue incidence rates, and most were not considered areas of high JEV endemicity, although some (eg, Bali, Indonesia) have recorded JEV cases and outbreaks in the past [11]. At the time of the study, Japanese encephalitis vaccine was not in routine use at most study sites [23].

In this study, we used the age-stratified serological data to describe JEV endemicity, and we estimated the force of infection (FOI) at the sites where this clinical trial was conducted. To account for cross-reactive, anti-flavivirus neutralizing antibodies, we stratified by dengue virus (DENV) serological status thereby providing minimal estimates of JEV infection in individuals who had never experienced a dengue infection; and higher estimates in those who have been infected with dengue in the past.

## METHODS

### Ethics Statement

This was a secondary analysis using records from a vaccine clinical trial, CYD14. The original clinical trial that generated the data (ClinicalTrials.gov number NCT01373281) underwent ethics committee approval of the protocol, amendments, consent, and assent forms and was funded by Sanofi Pasteur [22].

### Study Sample Set and Data

CYD14 was an observer-masked, randomized, controlled, multicenter, phase 3 dengue vaccine trial conducted in children aged 2–14 years old in 5 countries in the Asia-Pacific (3 sites in Indonesia [Bandung, Jakarta, and Bali]; 2 sites in Peninsular Malaysia [Kuala Lumpur and Penang]; 2 sites in the Philippines [San Pablo City and Cebu]; 2 sites in Thailand [Ratchaburi and Kamphaeng Phet]; and 2 sites in Southern Vietnam [My Tho and Long Xuyen]) and has been described previously [22]. Parents or legal guardians provided informed consent before participation, and written assent was obtained from older children, in compliance with the regulations of each country. Subjects received either 3 doses of a recombinant, live, attenuated, tetravalent dengue vaccine or placebo at months 0, 6, and 12.

In an immunological subset of approximately 20% of participants, serum was collected at baseline (before injection at month 0). Baseline concentrations of neutralizing antibody against JEV and DENV were measured by PRNT at the Centre for Vaccine Development (Mahidol Univeristy, Thailand) (for JEV) and at Sanofi Pasteur’s Global Clinical Immunology laboratory (Swiftwater, PA) (for DENV) using the method described by Timiryasova [24]. For DENV, challenge viruses were for DENV-1 strain PUO-359, DENV-2 strain PUO-218, DENV-3 strain PaH881/88, and DENV-4 strain 1228. Neutralizing antibody titers were expressed as the reciprocal serum dilution (1/dil) achieving 50% reduction in plaque count and a lower limit of quantification of ≥10, as calculated by probit analysis [25]. After an observation that JEV seroprevalence varied according to DENV serostatus, neutralizing JEV titers achieving 90% plaque reduction (PRNT_90_) were subsequently calculated from the same laboratory data to explore the impact of increasing specificity of the assay by decreasing the background serum cross-reactivity from other flaviviruses [21].

Individuals with a history of JEV or another flavivirus vaccination before blood sampling were removed from the analysis to ensure that serological status was a consequence of natural infection. Subjects from Thailand were excluded because Japanese encephalitis vaccination had been practiced nationwide for several years before the study and >95% of children were vaccinated, leaving a sample too small for meaningful analysis (n = 15).

### Japanese Encephalitis Virus Seroprevalence

Japanese encephalitis virus seroprevalence, defined as the proportion of subjects with a JEV-neutralizing antibody concentration of ≥10 (1/dil), was calculated according to PRNT_50_ and PRNT_90_, overall, and by age for each country. To control for the influence of cross-reactive DENV antibodies and generate a minimal estimate of true JEV-positive samples, JEV seroprevalence by PRNT_50_ was calculated separately for DENV seropositive and seronegative populations.

### Force of Infection

Catalytic models use seroprevalence data as cumulative markers of past infections that result in lifelong antibodies, from which force of primary infection estimates can be derived [26, 27]. An FOI model was developed to describe the rate of JEV infection over the period of time covered by the subjects’ age. The model assumed a constant FOI that does not vary with age whereby the probability of an individual being infected in 1 year is estimated by the following [28, 29]:

$${\text{p}}_{i}=\text{1}-{\text{e}}^{-\mu \text{A}i}$$

Where pi is the probability for the ith group of Ai years old of being positive and μ is the proportion of individuals infected per year, FOI. Using a maximum likelihood regression method, fitting a binomial model with a complementary log-log link function and using X = log(A) as an offset term, the intercept parameter α = log (µ) was estimated as follows:

Log (−log (1−pi))=log (μ)+log (Ai)

The exponential of α provided an estimate of the FOI, μ. Model fit was assessed using the Pearson and deviance test for goodness-of-fit statistics with a significance level of *P* < .05.

The proportion of individuals seropositive per age group, p_*i*_ was subsequently estimated with the following:

$$pi\;=\;1-e^{-\mu Ai}=1-e^{-(e^{\alpha }Ai)}$$

We considered that JEV serostatus in the DENV-naive population could not have been affected by cross-reactive flavivirus antibodies and therefore treated the resulting FOI estimate as a minimal estimate of annual infection risk.

All data were analyzed anonymously. Analyses were conducted using SAS 9.4, and figures were developed using Microsoft Excel 2013 and STATA, version 15.

## RESULTS

### Sample Set Description

**Figure 1.**
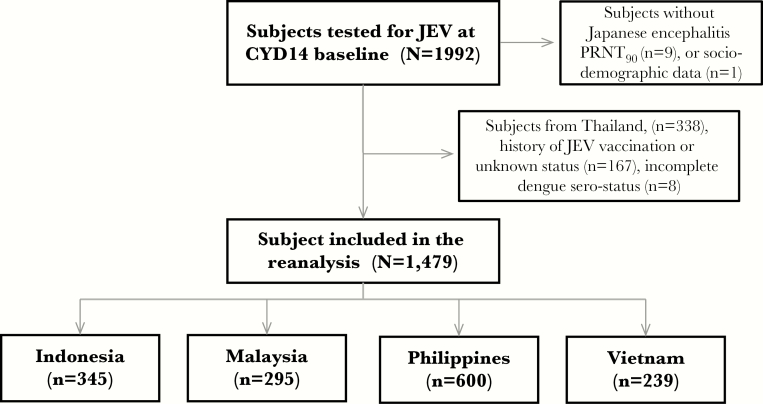
Study flow chart.

We conducted an epidemiological reanalysis of clinical trial data to document historical JEV exposure in 1479 children from 4 Asian countries, as shown in the study flow chart (Figure 1). The database included 239 subjects from Vietnam, 295 subjects from Malaysia, 345 subjects from Indonesia, and 600 subjects from the Philippines (Table 1). The mean age in each country was 8.24 in Indonesia, 8.25 in Malaysia, 8.18 in Philippines, and 7.55 in Vietnam.

**Table 1.** Number of Subjects Included by Age (N) and Japanese Encephalitis Seroprevalence by PRNT_50_ (%) According to DENV Serostatus

	Indonesia				Malaysia				Philippines				Vietnam			
Dengue Status	Positive		Negative		Positive		Negative		Positive		Negative		Positive		Negative	
Age	N	%	N	%	N	%	N	%	N	%	N	%	N	%	N	%
2	13	23%	11	9%	1	0%	21	0%	21	19%	18	22%	3	100%	7	14%
3	19	37%	10	0%	9	11%	12	0%	33	30%	24	8%	9	56%	11	46%
4	18	56%	13	8%	12	42%	12	0%	33	30%	20	10%	14	43%	8	38%
5	27	33%	6	0%	6	50%	25	8%	35	40%	17	6%	13	15%	22	9%
6	14	21%	2	0%	8	75%	10	0%	41	51%	14	7%	12	33%	10	30%
7	21	52%	2	50%	4	0%	9	11%	29	52%	6	0%	8	75%	4	25%
8	14	43%	3	0%	9	22%	10	10%	20	55%	5	20%	11	72%	8	38%
9	14	36%	3	0%	9	44%	9	11%	31	61%	4	25%	9	67%	6	50%
10	23	52%	3	0%	8	25%	4	0%	21	67%	1	0%	13	54%	7	43%
11	13	69%	2	50%	11	36%	8	0%	29	45%	-	-	12	75%	4	25%
12	51	75%	6	17%	30	57%	20	5%	58	64%	12	0%	28	71%	13	39%
13	34	74%	1	0%	24	42%	14	14%	68	75%	8	25%	6	100%	1	100%
14	22	73%	-	-	8	38%	2	50%	51	80%	1	0%	-	-	-	-
Total	283	54%	62	8%	139	41%	156	6%	470	55%	130	11%	138	59%	101	31%

Abbreviations: DENV, dengue virus; PRNT_50_, 50% plaque reduction neutralization test.

### Japanese Encephalitis Virus Seroprevalence and Rate of Infection

**Figure 2.**
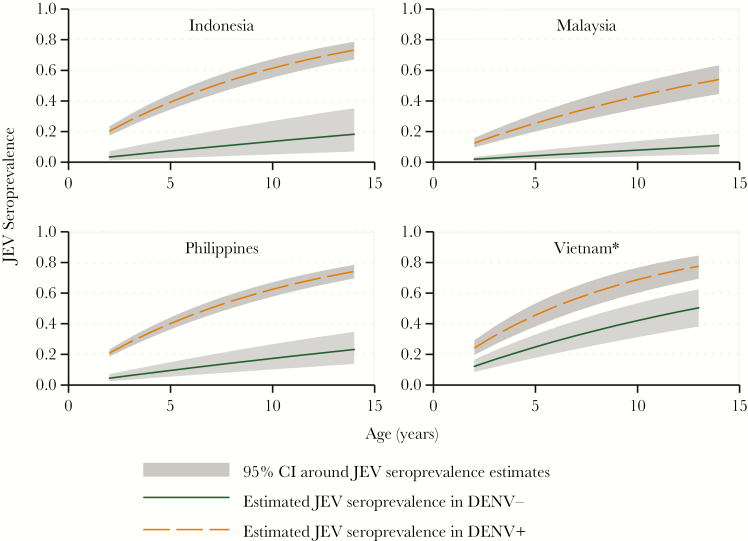
Force of infection-derived Japanese encephalitis age-specific seroprevalence estimates by country in dengue virus (DENV)-positive (DENV^+^) and DENV-negative (DENV^−^) subjects. *, Pearson and deviance test *P* < .05 for the DENV^+^ population in Vietnam. Abbreviations: CI, confidence interval; JEV, Japanese encephalitis virus.

By PRNT_50_, overall JEV seroprevalence was 46.1% in Indonesia, 22.4% in Malaysia, 45.7% in the Philippines, and 47.5% in Vietnam. Seroprevalence increased with age, reaching >70% in the 13- to 14-year-old children in Indonesia, the Philippines, and Vietnam and 40% in Malaysia (Table 1). When stratified by DENV serostatus, JEV seroprevalence was 54.4% in Indonesia, 41.0% in Malaysia, 55.3% in the Philippines, and 59.4% in Vietnam in DENV seropositive individuals and 8.1% in Indonesia, 5.8% in Malaysia, 10.8% in the Philippines, and 30.7% in Vietnam in DENV seronegative individuals. By JEV PRNT_90_, seroprevalence was considerably lower: 1.7% in Indonesia, 2.4% in Malaysia, 3.7% in the Philippines, and 11.3% in Vietnam. FOI estimates revealed an annual infection rate within DENV-positive subjects of 9.1% (95% confidence interval [CI], 7.7–10.7) in Indonesia, 5.4% (95% CI, 4.1–6.9) in Malaysia, 9.3% (95% CI, 8.2–10.6) in the Philippines, and 11.1% (95% CI, 8.8–13.8) in Vietnam. In DENV seronegative subjects, FOI was considerably lower: 1.4% (95% CI, 0.5–3.0) in Indonesia, 0.8% (95% CI, 0.4–1.4) in Malaysia, 1.8% (95% CI, 1.0–2.9) in the Philippines, and 5.2% (95% CI, 3.6–2.3) in Vietnam. The goodness-of-fit statistics were respected for all models (Pearson test, *P* > .05; deviance test, *P* > .05), except for the DENV-positive population in Vietnam (Figure 2).

## DISCUSSION

This study documented serological evidence of JEV circulation in urban and periurban areas of Indonesia, Malaysia, the Philippines, and Vietnam, countries with differing epidemiology and JEV risk. Our study assessed historical exposure to viruses but collected no data on symptomatic episodes. However, the World Health Organization’s vaccine-preventable disease monitoring system reports an annual average for these countries over recent years varying from 35.2 cases (Malaysia) to 310.6 cases (Vietnam) [30]. Even after correcting for a low proportion of symptomatic infections, the levels of pediatric infection documented here imply a significant level of underreporting of symptomatic cases. Measures to improve disease awareness and increase use of confirmatory diagnostics and surveillance enhancements may be justified in response.

Seroprevalence, a function of exposure, increased with age. Therefore, these age-stratified data allowed estimation of FOI, and, to our knowledge, this is the first time this has been done in a multicountry JEV study. JEV seroprevalence varied according to DENV status, which is likely a consequence of cross-reactive antibodies raised after DENV infections. Indeed, these sites were selected for their high levels of dengue endemicity, with annual attack rates of symptomatic dengue of 2%–11% per year [31]. Therefore, we estimated JEV FOI for individuals with no previous DENV exposure, resulting in minimal JEV exposure estimates, which provide strong evidence for JEV circulation within these study populations. By this measure, between 0.8% and 5.2% of children were estimated to be naturally infected by JEV annually, findings that may be considered high in areas that do not include JEV vaccination in their national immunization programs. The estimated JEV FOI in DENV-exposed individuals was considerably higher, and the true infection rate is likely somewhere in between. Although direct comparisons of JEV FOI are unavailable, historically, Japanese encephalitis has been a pediatric disease in endemic areas with seroprevalence increasing to 100% in adults [2].

For Vietnam, the goodness-of-fit statistics for the constant FOI risk model was statistically significant (*P* < .05), indicating that the assumption of constant risk of infection was not correct. This may be due to differential exposure at different ages or epidemic prone rather than endemic epidemiology. Vietnam is the country with highest infection risk, a finding aligned with the current knowledge of risk and epidemiology [32].

It is well known that flavivirus genera share epitopes that induce cross-reactive antibodies, which leads to difficulty in differentially diagnosing flaviviral infections [16, 17]. More recent or secondary infections generate broader, heterotypic, cross-reactive responses, and—because these sites were chosen due to their high level of DENV endemicity—we considered that anti-DENV antibodies would be more likely to cross-react with JEV virus in the PRNT than the reverse [16]. However, it is important to remember that cross-reactivity or neutralization does not mean cross-protection, and interactions between flavivirus antibodies are complex and poorly understood [17, 33]. Our additional observation that JEV seroprevalence by PRNT_50_ in DENV-naive children was higher than corresponding rates derived from JEV PRNT_90_ implies that PRNT_90_ is overly specific, excluding true-positive samples, for epidemiological studies such as this.

We assumed that the association between JEV and DENV serostatus was a product of cross-reactive antibodies, but this could also be caused by confounding by similar exposure risk to JEV and DENV. Japanese encephalitis virus and DENV are transmitted by different vector mosquitos, but behavioral or ecological risk factors such as increased outdoor exposure time or use of mosquito protective tools may predispose to risk of both [6, 34]. Well defined behavioral or ecological risk factors for these infections are poorly understood or lacking, and results of studies conducted across different geographies and time periods are seldom in agreement. In addition, although JEV infection in DENV-naive individuals provides confirmation of JEV circulation in a population with a low risk of flavivirus cross-reactivity, this may represent a specific population with less exposure to mosquito vectors, a selection bias that would underestimate true JEV transmission risk.

Limitations of this study include that subjects were not selected using a randomized or representative method and that, in the absence of virological confirmation of historical infections, it remains impossible to quantify the relative contribution of cross-reactivity in the PRNT assays. Interpretations cannot be generalized nationwide, and local experts and policymakers will need to decide the broader relevance of these findings for their countries.

## CONCLUSIONS

We report a clear demonstration of JEV infection risk and human transmission in regions of 4 countries. These regions were previously considered of low JEV risk and have no JEV vaccination programs in place.

## Notes

***Acknowledgments.*** We thank the children and their families who participated in the CYD14 study. We also thank Grenville Marsh of Sanofi Pasteur for editorial and manuscript coordination support.

***Financial support.*** This study required no external funding.

***Potential conflicts of interest.*** J. N., A.-F. T., A. M., M. B., D. C., and A. B. are employees of Sanofi Pasteur, a company engaged in the production of vaccines including against Japanese encephalitis and dengue viruses. S. Y., L. C. Q., M. R. C., S. R. H. and A. P. have been investigators for clinical or epidemiological studies sponsored by Sanofi Pasteur and have been remunerated accordingly. All authors have submitted the ICMJE Form for Disclosure of Potential Conflicts of Interest. Conflicts that the editors consider relevant to the content of the manuscript have been disclosed.

Present in part: 8th Asian Congress of Pediatric Infectious Diseases, November 2016, Bangkok, Thailand.
